# False-positive test results: prevalence, time trends, and factors associated with false-positive serological markers in blood donation screening

**DOI:** 10.1016/j.htct.2026.106499

**Published:** 2026-07-18

**Authors:** Lucas Kallas-Silva, Leandro Dinalli dos Santos, Valeria de Freitas Dutra, Jose Mauro Kutner, Carolina Bonet-Bub

**Affiliations:** a Faculdade Israelita de Ciencias da Saude Albert Einstein, Hospital Israelita Albert Einstein, São Paulo, Brazil; b Departamento Hemoterapia e Terapia Celular, Hospital Israelita Albert Einstein, São Paulo, Brazil

**Keywords:** Blood donors, Donor selection, Serologic tests, False-positive reactions, HIV

## Abstract

**Background and Objectives:**

False-positive serological results in blood donation screening may arise from antibody cross-reactivity, limitations in assay specificity, or low-level reactivity during early-stage infections, ultimately leading to donor deferral and the discard of viable blood units. This study evaluated the prevalence, time trends, and demographic factors associated with false-positive serologic screening results for transfusion-transmissible infections in a Brazilian blood center.

**Materials and Methods:**

A cross-sectional study was conducted including all blood donations collected between 2013 and 2022 at a private blood center in São Paulo, Brazil. Donations were screened with serologic tests for syphilis, human immunodeficiency virus, hepatitis B virus, and hepatitis C virus. Reactive samples underwent confirmatory testing, where a false-positive result was defined as an initially reactive screening assay followed by a nonreactive confirmatory test. Associations between demographic variables and false-positive or true-positive results compared to true negative results were analyzed using multivariable Poisson regression.

**Results:**

A total of 129,166 donors were included. Most donors (98.7%) had nonreactive results for all serologic tests. Reactive screening results were observed in 0.5% of donations for both syphilis and hepatitis B virus, 0.2% for hepatitis C virus, and 0.1% for human immunodeficiency virus. False positivity occurred more frequently for human immunodeficiency virus (92.0%) and hepatitis C virus (72.7%). Older age, lower education levels, and first-time donation were associated with false-positive results, whereas male sex, non-white race/skin color, lower education levels, and being single or divorced were associated with true-positive status.

**Conclusion:**

False-positive results were more frequent among first-time and less-educated donors. This study found no consistent overlap between demographic factors associated with false positivity and true positivity. This suggests that false positivity mainly occurs by chance or from antibody cross-reactivity.

## Introduction

Over the past decades, major advances in diagnostic assays for infectious diseases have improved the safety of blood donations. Currently, most blood collection organizations (BCOs) employ highly sensitive laboratory screening tests to detect transfusion-transmissible infections (TTIs), thereby minimizing the risk of transfusing contaminated blood products and making TTIs a rare event [1, 2, 3]. Globally, routine TTI screening typically includes tests for human immunodeficiency virus (HIV) [4,5], hepatitis B virus (HBV) [6], hepatitis C virus (HCV), human T-lymphotropic virus (HTLV) [7], and syphilis [8,9]. Additional testing for other TTIs includes Chagas disease, West Nile virus, Babesia, Malaria, and dengue, which are performed according to local epidemiology and risk assessment [10, 11, 12].

A major consequence of universal testing with highly sensitive diagnostic assays is the occurrence of false-positive results, which can have several undesirable implications. These include the unnecessary discard of safe blood units, increased laboratory workload and costs related to confirmatory testing, and significant emotional distress for donors, who may face permanent deferral or become reluctant to return for future donations [13, 14, 15].

False-positive serologic results may arise through several mechanisms. First, antibody cross-reactivity can occur in the presence of autoimmune disorders, heterophile antibodies, recent vaccinations, or unrelated infections [16,17]. Second, early-stage infections may present with incomplete antibody responses, leading to a reactive screening test but a non-reactive confirmatory result, thus interpreted as false positive [18, 19, 20]. Third, test-related technical factors, such as lot-to-lot variations or reduced assay specificity, can contribute to false reactivity [21]. Lastly, false-positive results may occur by chance, without a clearly identifiable underlying cause.

Previous studies have explored factors such as sex, educational attainment, and race/ethnicity as potential predictors of false reactivity in blood donation screening tests [13, 14, 15]. Although findings vary across infections and populations, some studies suggest that female sex, lower educational attainment, or African-American race and Hispanic ethnicity are associated with higher odds of false-positive results [13, 14, 15].

Despite these findings, recent data addressing how demographic factors influence the frequency of false-positive results using modern, high-performance assays remain limited. A clearer understanding of these associations could improve donor counseling, aid interpretation of discordant results, and encourage donors with previous false-positive findings to continue donating blood.

This study investigated the overall and annual frequencies of positive serological screening and false-positive serological results for syphilis, HIV, HCV, and HBV over a 10-year period at a blood center in São Paulo, Brazil. It also evaluated demographic factors associated with both false-positive and true-positive serologic screening results.

## Methods

### Study design

**Fig. 1:**
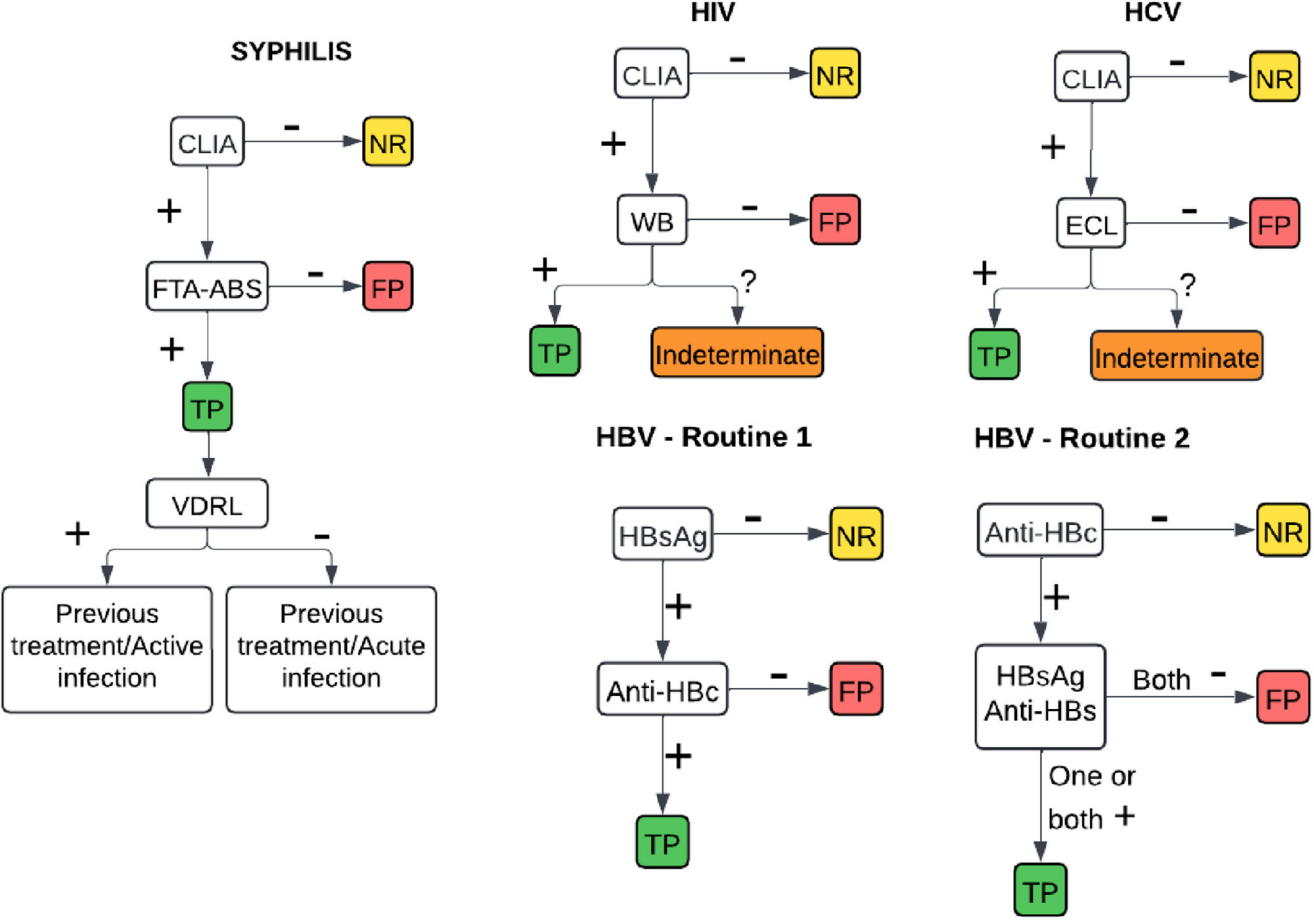
Screening routine and interpretations for syphilis, HIV, HBV, and HCV. NR: Non-reactive; FP: False positive; TP: True positive; CLIA: Chemiluminescent immunoassay; FTA-ABS: Fluorescent Treponemal Antibody Absorption; VDRL: Venereal Disease Research Laboratory test; WB: Western Blot; ECL: Electrochemiluminescence assay; HBsAg: Hepatitis B surface antigen; Anti-HBs: Hepatitis B surface antibody and Anti-HBc: Hepatitis B core antibody.

In this cross-sectional study, data from all blood donations collected between January 2013 and December 2022 at the BCO of Hospital Israelita Albert Einstein in São Paulo, Brazil, were analyzed. Demographic data, such as sex, age group, self-reported race/skin color, educational attainment, marital status and type of donation were retrieved from the institutional BCO database. For repeat donors, demographic data were extracted from their first recorded donation. Repeat donations were included independently of previous donations. All blood samples underwent routine screening for syphilis, HIV, HCV, and HBV according to institutional protocols focused on serological screening results (Figure 1). Nucleic Acid Testing (NAT) data for HIV, HCV, and HBV were also generated by the BCO but were excluded from this analysis.

### Screening routines

Screening for syphilis was performed using a chemiluminescent immunoassay (CLIA) (Alinity i Syphilis TP Reagent Kit; Abbott Diagnostics Division, Abbott Park, IL, USA). Reactive samples were subjected to confirmatory testing using the Fluorescent Treponemal Antibody Absorption assay (FTA-Abs; WAMA Diagnóstica, São Carlos, SP, Brazil) and the Venereal Disease Research Laboratory test (VDRL; WAMA Diagnóstica, São Carlos, SP, Brazil). False-positive results were defined as a reactive chemiluminescence immunoassay (CLIA) result accompanied by nonreactive FTA-Abs and VDRL results.

HIV screening was performed using CLIA-based methods. From 2013 to 2016, assays were conducted on the VIDAS platform (bioMérieux, Marcy-l’Étoile, France) and the VITROS platform (Ortho Clinical Diagnostics, Johnson & Johnson Company, Raritan, NJ, USA). Between 2017 and 2022, testing was changed to the Alinity i HIV Ag/Ab Combo Reagent Kit (Abbott Diagnostics Division, Abbott Park, IL, USA). Reactive results were confirmed using a Western Blot (WB) immunoassay (GS HIV-1 Western Blot; Bio-Rad Laboratories, Hercules, CA, USA). WB results were classified as reactive, indeterminate or nonreactive. A false-positive result was defined as a reactive CLIA result with a nonreactive WB result.

HCV screening was performed using a CLIA (Alinity i Anti-HCV Reagent Kit; Abbott Diagnostics Division, Abbott Park, IL, USA). Samples that tested reactive on CLIA underwent confirmatory testing using a recombinant immunoblot assay (RIBA) (Deciscan HCV PLUS; Bio-Rad Laboratories, Hercules, CA, USA). RIBA results were classified as reactive, indeterminate, or nonreactive. False-positive donations were defined as a reactive CLIA result with a nonreactive RIBA result.

HBV screening was performed using the Hepatitis B surface antigen (HBsAg) (Alinity i HBsAg Qualitative II Reagent Kit; Abbott Diagnostics Division, Abbott Park, IL, USA), Hepatitis B surface antibody (Anti-HBs) (Alinity i Anti-HBs Reagent Kit; Abbott Diagnostics Division, Abbott Park, IL, USA) and Hepatitis B core antibody (Anti-HBc) (Alinity i Anti-HBc Reagent Kit; Abbott Diagnostics Division, Abbott Park, IL, USA). A false-positive result was defined under two conditions: (1) Reactive HBsAg with nonreactive anti-HBc; or (2) Reactive anti-HBc with nonreactive HBsAg and nonreactive anti-HBs. If either HBsAg or anti-HBs results were reactive, the donation was considered a true positive.

All donations with positive screening results for any infection were immediately discarded according to institutional policy. Donors with false-positive screening results were invited for retesting at approximately one month and five months after the initial donation to assess potential seroconversion. Donors with confirmed seroconversion were referred for clinical follow-up. Those with persistently negative results during the follow-up were eligible for reintegration into the donor pool.

### Statistical analysis

Demographic characteristics of donors were described by screening result category. Age was categorized into five groups (≤25, 26–35, 36–45, 46–55, and >55 years). Educational attainment was categorized into three groups (complete college or higher education, incomplete college, and high school or less). Differences between groups were assessed using the chi-squared test. Frequencies and percentages of reactive and false-positive results were calculated and reported as annual distributions. Associations between demographic factors and false-positive screening results (versus nonreactive results) were analyzed using univariable and multivariable Poisson regression models. Variables included in the multivariable model were sex, age category, self-reported race/skin color, educational level, marital status, and type of donation. Similar analyses were performed for true-positive results when the number of cases permitted. Regression results were presented as prevalence ratios (PRs) with 95% confidence intervals (95% CIs) and p-values.

Analyses were performed separately for each infection and for grouped screening routines. Flowcharts illustrating the testing procedures were generated using Lucidchart (https://www.lucidchart.com). All statistical analyses were conducted in R software, version 4.3.1 (R Foundation for Statistical Computing, Vienna, Austria).

### Ethical considerations

An Ethics Review Board revised and approved the study (Hospital Israelita Albert Einstein, CAAE Number 61,849,622.3.0000.0071) with no requirement of additional consent for use of de-identified donation data. The study performed no additional tests on donated blood and collected no additional information from participants. The manuscript followed the Strengthening the Reporting of Observational Studies in Epidemiology (STROBE) guidelines [22].

## Results

### Participants

Between January 2013 and December 2022, a total of 129,166 donations were received and underwent infectious disease screening at the Hospital Israelita Albert Einstein BCO. Table 1 summarizes the demographic characteristics of participants according to serologic screening results. The median age of donors was 37 years (interquartile range [IQR]: 28.0–46.6). Among all participants, 51,543 (39.9%) were female, 95,065 (73.6%) self-identified as White, 79,313 (61.4%) reported a college-level education or higher, and 56,569 (43.8%) were single. Regarding donation history, 71,808 (56.0%) were classified as repeat donors (≥2 donations within 12 months) or sporadic donors (1 donation every 12 months).

**Table 1:** Characteristics of study participants, overall and by screening result, independent of confirmation tests results.

	**HBV**	**HCV**	**HIV**	**Syphilis**	**TN**
	**n=614**	**n=292**	**n=126**	**n=677**	**n=127,457**
**Age – years (%)**					
≤25	75 (12.2%)	55 (18.8%)	21 (16.7%)	117 (17.3%)	20,718 (16.3%)
26–35	124 (20.2%)	80 (27.4%)	41 (32.5%)	208 (30.7%)	33,736 (26.5%)
36–45	173 (28.2%)	74 (25.3%)	35 (27.8%)	159 (23.5%)	35,474 (27.8%)
46–55	170 (27.7%)	48 (16.4%)	20 (15.9%)	110 (16.2%)	24,801 (19.5%)
>55	72 (11.7%)	35 (12.0%)	9 (7.1%)	83 (12.3%)	12,728 (10.0%)
**Sex (%)**					
Female	261 (42.5%)	140 (47.9%)	63 (50.0%)	259 (38.3%)	50,820 (39.9%)
Male	353 (57.5%)	152 (52.1%)	63 (50.0%)	418 (61.7%)	76,637 (60.1%)
**Race/Skin color (%)**					
Non-White	175 (28.5%)	82 (28.1%)	35 (27.8%)	219 (32.3%)	33,196 (26.0%)
White	436 (71.0%)	210 (71.9%)	91 (72.2%)	454 (67.1%)	93,874 (73.7%)
**Highest education level (%)**					
Complete college or higher	324 (52.8%)	158 (54.1%)	69 (54.8%)	338 (49.9%)	78,424 (61.5%)
Incomplete college	43 (7.0%)	35 (12.0%)	14 (11.1%)	78 (11.5%)	14,552 (11.4%)
High school or less	241 (39.3%)	94 (32.2%)	42 (33.3%)	248 (36.6%)	32,618 (26.0%)
**Marital Status (%)**					
Divorced/Separated	59 (9.6%)	21 (7.2%)	12 (9.5%)	67 (9.9%)	7886 (6.2%)
Married/Civil Union	316 (51.5%)	140 (47.9%)	62 (49.2%)	258 (38.1%)	62,543 (49.1%)
Single	229 (37.3%)	130 (44.5%)	52 (41.3%)	346 (51.1%)	55,812 (43.8%)
**Type of donation (%)**					
First time	557 (90.7%)	232 (79%)	91 (72.2%)	549 (81.1%)	55,905 (43.9%)
Repeat/Sporadic	57 (9.3%)	60 (21%)	35 (27.8%)	128 (18.9%)	71,552 (56.1%)

TN: True Negative; HBV: Hepatitis B Virus; HCV: Hepatitis C Virus; HIV: Human Immunodeficiency Virus.

### Screening and temporal trends in false-positive results

Overall, 127,457 (98.7%) donations had negative screening results for all infections. During the study period, positive screening results were observed in 677 (0.5%; range: 0.3%−0.8%) for syphilis, 126 (0.1%; range: 0.02%−0.2%) for HIV; 292 (0.2%; range: 0.1%−0.3%) for HCV and 614 (0.5%; range: 0.3%−0.7%) for HBV.

**Fig. 2:**
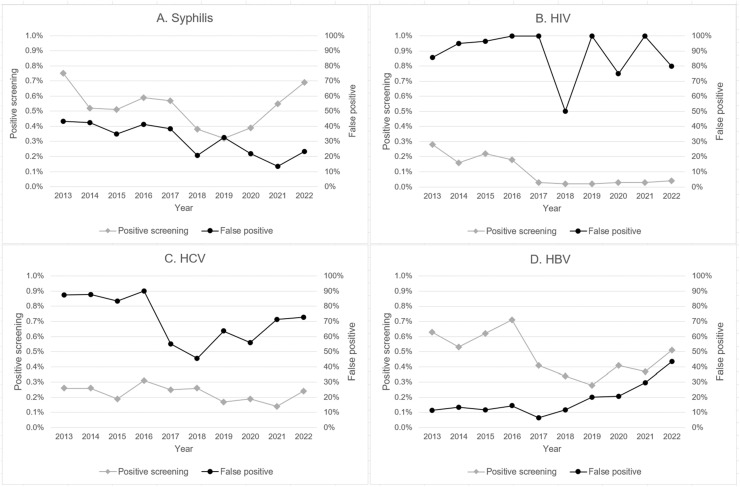
Screening and false positive results by year. HBV: Hepatitis B Virus; HCV: Hepatitis C Virus; HIV: Human Immunodeficiency Virus.

During the study period, among participants with positive screening results, 214 (31.6%; range: 13.6%−43.7%) were false positive for syphilis, 126 (92.0%; range: 80.0%−100%) for HIV, 211 (72.7%; range: 45.5%−90.0%) for HCV and 112 (18.2%; range: 6.4%−43.5%) for HBV. The annual frequencies of reactive and false-positive results for each infection are presented in Figure 2. This study identified a decrease in syphilis-reactive screening results from 2013 to 2019, followed by a gradual increase through 2022. False-positive results for syphilis declined until 2018 but remained stable thereafter. For HIV, a marked decline in screening reactivity occurred in 2017 and then remained stable until 2022. HCV screening frequencies remained relatively constant throughout the study period, although false-positive results decreased steadily from 2017 to 2022. HBV screening reactivity decreased between 2017 and 2019 and subsequently stabilized, whereas false-positive HBV results increased slightly after 2018.

### Poisson regression models assessing factors associated with false-positive and true-positive results

Table 2, Table 3, Table 4, Table 5, Table 6 show the results of univariable and multivariable models assessing factors associated with false-positive results compared to true negative results. Table 2, Table 3, Table 4 show the results of univariable and multivariable models assessing factors associated with true-positive results compared to true negative results for syphilis and HBV. Poisson models for true-positive results were not conducted for HIV and HCV due to the insufficient sample size.

**Table 2:** Univariable and multivariable models addressing factors associated with false-positive and true-positive results for syphilis, HIV, HBV, or HCV.

	**FP - PR**	**FP**			**TP**	
	**(95% CI)**	**p-value**	**aPR (95% CI)**	**p-value**	**aPR (95% CI)**	**p-value**
**Sex**						
Female	Reference	—	Reference	—	Reference	—
Male	0.82 (0.70–0.96)	0.011	0.95 (0.81–1.11)	0.491	1.33 (1.17–1.52)	<0.001
**Age category (years)**						
≤25	Reference	—	Reference	—	Reference	—
26–35	0.92 (0.73–1.18)	0.517	1.25 (0.97–1.63)	0.088	1.79 (1.44–2.23)	<0.001
36–45	1.01 (0.80–1.27)	0.948	1.53 (1.15–2.04)	0.004	2.01 (1.58–2.57)	<0.001
46–55	0.78 (0.60–1.02)	0.073	1.30 (0.94–1.81)	0.114	3.44 (2.67–4.43)	<0.001
>55	0.95 (0.70–1.28)	0.746	1.72 (1.18–2.48)	0.004	4.02 (3.00–5.37)	<0.001
self-reported race/skin color						
White	Reference	—	Reference	—	Reference	—
Non-white	1.06 (0.89–1.25)	0.523	0.95 (0.80–1.14)	0.609	1.15 (1.00–1.32)	0.049
**Education**						
Complete college or higher	Reference	—	Reference	—	Reference	—
Incomplete college	1.32 (1.04–1.67)	0.021	1.49 (1.14–1.92)	0.003	1.01 (0.78–1.29)	0.948
≤ High school	1.45 (1.22–1.72)	<0.001	1.49 (1.24–1.78)	<0.001	1.84 (1.59–2.11)	<0.001
**Marital status**						
Married/Civil Union	Reference	—	Reference	—	Reference	—
Divorced/Separated	1.00 (0.71–1.37)	0.983	1.00 (0.71–1.37)	0.989	2.03 (1.63–2.52)	<0.001
Single	0.97 (0.83–1.14)	0.696	0.95 (0.78–1.16)	0.625	1.63 (1.38–1.91)	<0.001
**Type of donation**						
Repeat/Sporadic	Reference	—	Reference	—	Reference	—
First time	3.54 (2.98–4.22)	<0.001	3.57 (2.99–4.29)	<0.001	14.5 (11.7–18.3)	<0.001

FP: False positive; TP: True positive; PR: Prevalence Ratio; aPR: Adjusted Prevalence Ratio; CI: Confidence Interval; HBV: Hepatitis B Virus; HCV: Hepatitis C Virus; HIV: Human Immunodeficiency Virus.

**Table 3:** Univariable and multivariable models addressing factors associated with false-positive and true-positive syphilis serologies.

	**FP - PR**	**FP**			**TP**	
	**(95% CI)**	**p-value**	**aPR (95% CI)**	**p-value**	**aPR (95% CI)**	**p-value**
**Sex**						
Female	Reference	—	Reference	—	Reference	—
Male	0.97 (0.74–1.28)	0.816	1.09 (0.82–1.45)	0.557	1.51 (1.25–1.84)	<0.001
**Age category (years)**						
≤25	Reference	—	Reference	—	Reference	—
26–35	1.12 (0.71–1.80)	0.634	1.51 (0.92–2.52)	0.105	1.83 (1.38–2.42)	<0.001
36–45	1.60 (1.05–2.51)	0.032	2.43 (1.44–4.17)	<0.001	1.34 (0.95–1.89)	0.096
46–55	1.01 (0.62–1.68)	0.955	1.67 (0.91–3.08)	0.100	1.99 (1.37–2.89)	<0.001
>55	1.39 (0.80–2.40)	0.232	2.66 (1.38–5.12)	0.003	3.65 (2.43–5.44)	<0.001
self-reported race/skin color						
White	Reference	—	Reference	—	Reference	—
Non-white	0.93 (0.68–1.26)	0.652	0.84 (0.60–1.16)	0.308	1.34 (1.09–1.63)	0.004
**Education**						
Complete college or higher	Reference	—	Reference	—	Reference	—
Incomplete college	1.20 (0.78–1.79)	0.387	1.61 (1.00–2.49)	0.040	1.20 (0.86–1.65)	0.266
≤ High school	1.23 (0.90–1.67)	0.182	1.36 (0.97–1.87)	0.066	1.81 (1.47–2.23)	<0.001
**Marital status**						
Married/Civil Union	Reference	—	Reference	—	Reference	—
Divorced/Separated	0.91 (0.49–1.56)	0.755	0.93 (0.50–1.60)	0.810	3.05 (2.19–4.17)	<0.001
Single	0.84 (0.63–1.12)	0.235	0.99 (0.70–1.40)	0.974	2.20 (1.72–2.82)	<0.001
**Type of donation**						
Repeat/Sporadic	Reference	—	Reference	—	Reference	—
First time	2.74 (2.07–3.68)	<0.001	2.98 (2.22–4.05)	<0.001	9.47 (7.22–12.7)	<0.001

FP: False positive; TP: True positive; PR: Prevalence Ratio; aPR: Adjusted prevalence Ratio; CI: Confidence Interval.

**Table 4:** Univariable and multivariable models addressing factors associated with false-positive and true-positive HBV serologies.

	**FP - PR**	**FP**			**TP**	
	**(95% CI)**	**p-value**	**aPR (95% CI)**	**p-value**	**aPR (95% CI)**	**p-value**
**Sex**						
Female	Reference	—	Reference	—	Reference	—
Male	1.02 (0.70–1.51)	0.899	1.26 (0.85–1.88)	0.252	1.17 (0.97–1.40)	0.100
**Age category (years)**						
≤25	Reference	—	Reference	—	Reference	—
26–35	0.57 (0.32–0.99)	0.045	0.67 (0.36–1.23)	0.198	1.85 (1.30–2.67)	<0.001
36–45	0.72 (0.43–1.22)	0.212	0.87 (0.44–1.71)	0.688	3.11 (2.16–4.53)	<0.001
46–55	0.64 (0.35–1.15)	0.137	0.85 (0.40–1.81)	0.684	5.67 (3.91–8.31)	<0.001
>55	0.63 (0.29–1.26)	0.209	0.64 (0.23–1.65)	0.378	4.60 (2.95–7.17)	<0.001
self-reported race/skin color						
White	Reference	—	Reference	—	Reference	—
Non-white	0.94 (0.60–1.43)	0.787	0.86 (0.55–1.32)	0.506	1.00 (0.82–1.22)	0.965
**Education**						
Complete college or higher	Reference	—	Reference	—	Reference	—
Incomplete college	1.55 (0.88–2.60)	0.110	1.53 (0.81–2.74)	0.168	0.80 (0.51–1.19)	0.290
≤ High school	1.43 (0.93–2.15)	0.096	1.33 (0.84–2.08)	0.214	1.94 (1.60–2.35)	<0.001
**Marital status**						
Married/Civil Union	Reference	—	Reference	—	Reference	—
Divorced/Separated	0.82 (0.32–1.75)	0.645	0.74 (0.26–1.68)	0.516	1.52 (1.11–2.04)	0.007
Single	0.89 (0.60–1.31)	0.551	0.70 (0.42–1.16)	0.177	1.29 (1.03–1.61)	0.028
**Type of donation**						
Repeat/Sporadic	Reference	—	Reference	—	Reference	—
First time	4.02 (2.65–6.32)	<0.001	3.91 (2.54–6.22)	<0.001	25.2 (17.5–37.8)	<0.001

FP: False positive; TP: True positive; PR: Prevalence Ratio; aPR: Adjusted prevalence Ratio; CI: Confidence Interval; HBV: Hepatitis B Virus.

**Table 5:** Univariable and multivariable models addressing factors associated with false-positive HIV serology.

	**PR (95% CI)**	**p-value**	**aPR (95% CI)**	**p-value**
**Sex**				
Female	Reference	—	Reference	—
Male	0.60 (0.41–0.86)	0.006	0.68 (0.47–0.99)	0.044
**Age category (years)**				
≤25	Reference	—	Reference	—
26–35	1.20 (0.70–2.12)	0.527	1.64 (0.91–3.03)	0.108
36–45	1.01 (0.58–1.82)	0.961	1.52 (0.77–3.02)	0.229
46–55	0.84 (0.44–1.59)	0.580	1.25 (0.57–2.72)	0.570
>55	0.69 (0.28–1.51)	0.370	1.17 (0.43–2.93)	0.747
Self-reported race/skin color				
White	Reference	—	Reference	—
Non-white	1.08 (0.71–1.60)	0.720	0.90 (0.58–1.36)	0.631
**Education**				
Complete college or higher	Reference	—	Reference	—
Incomplete college	1.28 (0.69–2.22)	0.409	1.43 (0.74–2.61)	0.258
≤ High school	1.71 (1.15–2.53)	0.008	1.87 (1.23–2.84)	0.003
**Marital status**				
Married/Civil Union	Reference	—	Reference	—
Divorced/Separated	1.61 (0.80–2.97)	0.147	1.68 (0.82–3.12)	0.124
Single	1.06 (0.72–1.55)	0.772	0.93 (0.58–1.47)	0.755
**Type of donation**				
Repeat/Sporadic	Reference	—	Reference	—
First time	3.22 (2.17–4.88)	<0.001	3.08 (2.05–4.74)	<0.001

PR: Prevalence Ratio; aPR: Adjusted prevalence Ratio; CI: Confidence Interval; HIV: Human Immunodeficiency Virus.

**Table 6:** Univariable and multivariable models addressing factors associated with falsely reactive HCV serology.

	**PR (95% CI)**	**p-value**	**aPR (95% CI)**	**p-value**
**Sex**				
Female	Reference	—	Reference	—
Male	0.74 (0.56–0.97)	0.026	0.86 (0.65–1.14)	0.293
**Age category (years)**				
≤25	Reference	—	Reference	—
26–35	0.90 (0.61–1.35)	0.599	1.32 (0.85–2.04)	0.215
36–45	0.76 (0.50–1.14)	0.177	1.31 (0.80–2.16)	0.288
46–55	0.67 (0.42–1.06)	0.090	1.34 (0.76–2.35)	0.304
>55	0.95 (0.57–1.56)	0.851	2.18 (1.16–4.01)	0.013
Self-reported race/skin color				
White	Reference	—	Reference	—
Non-white	1.23 (0.91–1.64)	0.166	1.13 (0.83–1.52)	0.428
**Education**				
Complete college or higher	Reference	—	Reference	—
Incomplete college	1.40 (0.90–2.08)	0.115	1.44 (0.90–2.23)	0.115
≤ High school	1.58 (1.17–2.13)	0.003	1.53 (1.11–2.10)	0.009
**Marital status**				
Married/Civil Union	Reference	—	Reference	—
Divorced/Separated	0.87 (0.44–1.55)	0.668	0.86 (0.43–1.54)	0.635
Single	1.12 (0.85–1.48)	0.422	1.07 (0.75–1.51)	0.719
**Type of donation**				
Repeat/Sporadic	Reference	—	Reference	—
First time	4.71 (3.42–6.62)	<0.001	4.60 (3.31–6.53)	<0.001

PR: Prevalence Ratio; aPR: Adjusted prevalence Ratio; CI: Confidence Interval; HCV: Hepatitis C Virus.

In the multivariable Poisson regression model assessing factors associated with false-positive results across all serologic tests, being aged 36–45 years or >55 years was significantly associated with false positivity compared with ≤25 years. Lower education was associated with a higher likelihood of false-positive results. Additionally, first-time donors were more likely to present false-positive reactivity compared with repeat donors.

In the multivariable model evaluating factors associated with true-positive results across all infections, male sex, older age, non-white race/skin color, lower education, single or divorced/separated status, and first-time donor status were all significantly associated with this outcome (Table 2).

In the model examining factors associated with false-positive results for syphilis, being aged 36–45 years or >55 years, having incomplete college education, and being a first-time donor were independently associated with false positivity. In contrast, for true-positive syphilis results, male sex and older age (except for the 36–45-year group) were associated with true positivity. Additionally, non-white race/skin color, low educational attainment, single or divorced/separated status, and first-time donation were associated with true-positive outcomes (Table 3).

For false-positive HBV results, first-time donation was significantly associated with increased likelihood of false positivity. For true-positive HBV results, all age categories above 25 years, lower educational attainment, single or divorced/separated status, and first-time donation were significantly associated with true positivity (Table 4).

In the model evaluating HIV testing, being of the female sex, having only completed high school or less and being a first-time donor were associated with false-positive results (Table 5).

In the model addressing false-positive results for HCV testing, being aged >55 years, having only high school education or less and being a first-time donor were associated with false-positive results (Table 6).

## Discussion

Data from >120,000 blood donors were analyzed in this 10-year cross-sectional study conducted at a major blood center in São Paulo, Brazil, to assess annual trends in positive screening and false-positive results for syphilis, HIV, HCV, and HBV. Demographic factors associated with false-positive and true-positive results were also examined. Nearly all donations had nonreactive screening results (98.7%), with reactive results most frequent for syphilis (0.5%) and HBV (0.5%). Among positive screening tests, false positivity was most common for HIV (92.0%) and HCV (72.7%).

As for temporal trends, given the relatively small number of reactive samples, year-to-year variability likely reflects random variations rather than true epidemiologic shifts, when considering positive serology and false-positive results for syphilis, HBV, and HCV. For HIV, a marked decline in screening reactivity occurred in 2017 and remained stable until 2022, likely reflecting the replacement of the bioMérieux CLIA platform and Ortho Clinical Diagnostics with assays from Abbott Diagnostics that year. Decreases in false-positive HIV results in 2018, 2020, and 2022 further support an assay-related effect.

In the multivariable Poisson regression models, female sex was associated with a higher prevalence of false-positive results in the HIV model, consistent with previous studies [14]. This pattern may be attributable to higher frequencies of autoantibodies and nonspecific antibody reactivity among women [23]. Such cross-reactivity is a known source of false-positive serologic findings [16,17,24].

Older age was also associated with false-positive results in the overall, syphilis and HCV models though not consistently across infections. This finding aligns with previous research suggesting that older individuals may produce higher titers of nonspecific antibodies after immune stimulation, which can increase assay cross-reactivity [25].

Lower educational attainment was another consistent predictor of false-positive results across most analyses. This association may indirectly reflect socioeconomic status: individuals with lower education may be more exposed to infections or immunologic stimuli, potentially increasing antibody cross-reactivity [13,26]. Lower educational attainment was also associated with true positivity, which may reflect a lower access to healthcare and diagnostic testing [27]. Importantly, limited health literacy associated with lower educational attainment [28] may influence the donor’s ability to understand the meaning and implications of false-positive results, potentially increasing the risk of misinterpretation and emotional distress if results are not clearly explained.

First-time donors demonstrated the strongest and most consistent association with false-positive results. Similar findings have been reported previously [7,13,14]. This association may be partially influenced by selection bias, as donors with false-positive results may be discouraged from future donations. Repeat and sporadic donors who have previously tested negative may represent a self-selected population less likely to present false reactivity, resulting in a repeat donor population with inherently lower reactivity rates. First-time donor status was also consistently associated with true-positive results, likely driven by a similar structural mechanism: donors with prior true-positive results are permanently deferred from future donation. Lastly, another possible mechanism is associated with risk behaviors: studies show that repeat donors have fewer high-risk behaviors when compared to first-time donors [29]. Return rates among donors with false-positive results were not evaluated, which could provide further insight into donor retention and behavioral responses following reactive screening.

No significant associations were found between race/skin color or marital status and false-positive results in this study although other reports have described higher false reactivity among non-White donors [7,13,14]. Such discrepancies may reflect regional demographic differences or variations in assay performance.

Due to the small number of true-positive cases, analyses to assess factors associated with true-positive results were not conducted for HIV and HCV. In the overall, syphilis and HBV models, male sex and older age were associated with higher prevalence of true-positive results, consistent with known epidemiologic patterns of TTIs. Non-white race/skin color was associated with true positivity in the overall and syphilis models but not for HBV. Across all models, lower educational attainment, being single or divorced/separated and first-time donation were consistently related to true positivity.

These findings reinforce the importance of demographic and behavioral screening in donor selection, while highlighting the balance between high assay sensitivity and the occurrence of nonspecific reactivity in maintaining transfusion safety. However, the lack of consistent overlap between variables associated with false-positive and true-positive results suggests that the mechanisms underlying false reactivity differ from those driving true infection risk.

False-positive serologic results are commonly attributed to nonspecific antibody cross-reactivity, which may occur following vaccination, infection, allergic reactions, autoimmune processes or exposure to transplantation antigens [16,17,24]. Differences in assay design and antigen composition can also influence specificity and lead to spurious reactivity [30].

The observed association between female sex and HIV false positivity may reflect immunologic cross-reactivity due to the higher prevalence of autoantibodies in women compared to men [23]. Similarly, the association between lower education and false reactivity may indicate increased immune stimulation from exposure to infections in lower socioeconomic contexts [26]. Older individuals may have accumulated immunologic experiences or demonstrate more pronounced polyclonal antibody responses, contributing to the age-related trend [25].

Although false positivity can also result from incomplete antibody responses during early infection - where screening is reactive but confirmatory testing is negative [18, 19, 20] - the lack of consistent overlap between predictors of false and true positivity in the present study suggests that this mechanism was not the predominant cause.

This study has several limitations. Results from subsequent donations of participants who experienced prior false-positive outcomes were not compared. This omission limits the possibility to analyze the underlying mechanisms that drive these occurrences. On false-positive results, additional testing was not conducted to exclude incipient infections, neither was testing to explore cross-reactivity. Although nucleic acid testing (NAT) is routinely performed in the BCO, these data were not included, and some cases classified as false positive may represent early window-period infections. However, given the low prevalence of such window-period infections, this potential misclassification is unlikely to substantially affect the overall findings [31]. Moreover, the change in HIV screening assays in 2017 led to an immediate reduction in reactivity rates, which may limit the interpretation of time trends for this infection. No similar assay transitions occurred for the other infections. Additionally, the study did not collect behavioral or health-related variables, such as sexual activity, history of infections or chronic diseases, which could influence false-positive rates. Nonetheless, this large ten-year dataset provides a robust overview of serologic reactivity trends and associated factors among Brazilian blood donors.

## Conclusions

The findings of this study demonstrate that certain demographic characteristics, particularly first-time donation and lower educational attainment, are consistently associated with a higher prevalence of false-positive serologic results in blood donor screening. Older age showed a weaker and less consistent association. The lack of overlap between predictors of false-positive and true-positive results supports the hypothesis that most false-positive findings arise from cross-reactive antibody mechanisms rather than incomplete antibody responses.

These results underscore the need for further studies investigating immune and technical mechanisms underlying false reactivity in blood donation screening. Future research should also explore the psychological and operational impacts of false-positive results and evaluate strategies to support donor retention following false reactivity notifications.

## Funding

The authors received no specific funding for this work

## Data availability

The data that support the findings of this study are available from the corresponding author upon reasonable request.

## Author contributions

L.K.S. performed the research, analyzed the data, wrote the first draft of the manuscript and reviewed and edited the manuscript. L.D.S. collected data, designed the study and reviewed and edited the manuscript. V.F.D. collected data, designed the study, supervised the research and reviewed and edited the manuscript. J.M.K. collected data, supervised the research and reviewed and edited the manuscript. C.B.B. designed the study, supervised the research and reviewed and edited the manuscript.

## Conflicts of interest

The authors have no conflicts of interest to declare.
